# Prevalence and co-occurrence of psychiatric symptom clusters in the U.S. adolescent population using DISC predictive scales

**DOI:** 10.1186/1745-0179-1-22

**Published:** 2005-10-28

**Authors:** Kevin W Chen, Ley A Killeya-Jones, William A Vega

**Affiliations:** 1 Department of Psychiatry, University of Medicine and Dentistry of New Jersey – Robert Wood Johnson Medical School, 671 Hoes Lane, Piscataway, New Jersey 08854, USA; 2 Center for Child and Family Policy, Duke University, Box 90545, Durham, NC 27708, USA

**Keywords:** adolescent, mental health, symptom clusters, comorbidity, DISC predictive scale, demographic correlates.

## Abstract

**Objective:**

To estimate 12-month prevalence and co-occurrence of symptoms of specific mental problems among US adolescents (12–17 years) by age, sex and racial/ethnic subgroups.

**Method:**

Data from the 2000 National Household Survey of Drug Abuse (NHSDA) adolescent sample are used to estimate prevalence and co-occurrence rates using the DISC predictive scales. Multiple logistic regressions were used to derive significant correlates of each domain of DPS-derived symptom cluster indicators of psychiatric problems and of severe comorbidity, with control of demographics and environmental factors.

**Setting:**

The National Household Survey on Drug Abuse (NHSDA), a national household probability sample, includes a nationally representative sample of 12–17 year-old adolescents (N = 19,430), through in-home surveys.

**Results:**

Three out of five adolescents screened positive for at least one DPS symptom cluster with estimates for specific symptom cluster ranging over 9.7% (substance use disorder), 13.4% (affective), 36.3% (disruptive-behavior), and 40.1% (anxiety). Co-occurrence was high with almost one-third of any DPS symptom cluster reporting multiple positive screens of four or more clusters. Blacks and younger females were most likely to report mental health problems and co-occurrence.

**Conclusion:**

Mental health problems among U.S. youth may be far more common than previously believed, although these symptoms have not yet reached the point of clinical impairment. The data speak to important patterns of age, gender and racial/ethnic differences in mental health problems deserving of further study.

## Introduction

Although mental health has become increasingly important in our healthcare model and definition of health, especially during childhood and adolescence[1,2], little is known about actual prevalence of various psychiatric disorders among adolescents in the general population. Current estimates range from 10% to 60% [3-5], based mostly on local community or treatment samples and dependent upon the case-finding protocol used. Evidence from adult samples suggests that many disorders with psychiatric symptoms begin during childhood or adolescence[6,7], although estimates of the earlier onset of adult disorders may be unreliable due to errors in retrospective recall[8]. It is well documented that childhood and adolescent mental health problems have profound implications for negative adult sequelae [9-13]. By official estimate, at any one time, about 20% of US children and adolescents have at least one diagnosable mental health disorder[1]. However, in their longitudinal study of 9–16 year-old adolescents, Costello et al. reported 36.7% having at least one psychiatric disorder (by DSM-IV criteria) during the study period (although the prevalence of a number of disorders dropped precipitously by age 12)[4]. Turner & Gil reported that 60% of their community youth sample (age 19–21) met lifetime criteria for one or more mental disorders, including substance use disorders (SUD), with full DSM-IV diagnosis[5].

Recent national surveys provided some important epidemiological information about the prevalence of psychiatric disorders among adults [14-18], but our understanding of the scope of psychiatric disorders or symptoms among children and adolescents is limited by a number of methodological constraints, including small samples, or samples from clinics or institutions; overly specific research foci; and screening questions either limited in number or not closely aligned with DSM diagnostic criteria. Thus, the estimates of prevalence in the general U.S. adolescent population cannot be reliably made, presenting a major obstacle for estimating the course and magnitude of adolescents' mental health problems, the degree of both need and *unmet *need, and for developing effective prevention and intervention programs for this critical age group[4,19] Estimates from adult national probability samples suggest that nearly half of all adult cases report onset by age 14, and three-quarters by age 24[6,18]. However, it is not clear what major types of mental health problems adolescents are confronted with, and to what extent. Moreover, there is a lack of ethnic group-specific estimates of mental health needs that would be required for more targeted services for various subpopulations. Importantly, members of racial/ethnic minority groups report some of the highest rates of unmet need for treatment as adults[20,21].

One way to provide uniform estimates of mental problems would be to include in national surveys of the general population structured diagnostic interviews or selected screening items or scales of symptoms of psychiatric problems that have high predictive value for diagnosis. Although such a survey cannot constitute an actual diagnosis of disorder, it can facilitate identification of groups at high risk, as well as help elucidate differential patterns in important demographic groups, including age, gender and race/ethnicity. An important caveat of using this approach to estimate psychiatric symptoms among adolescents is that this is a group experiencing profound developmental changes across biological, psychological and social domains[2]. Thus, there remains some question of the extent to which psychiatric symptoms are stable indicators of some underlying need or indicative of a developing disorder[22], or whether they are indicative of normal (vs. problematic) behavior[23]. Moreover, cross-sectional surveys can only present a snapshot of prevalence estimates; changes over time and the ways in which such symptoms evolve into diagnosable disorders cannot be measured. Several empirical studies based on the Great Smokey Mountains Study[24] have demonstrated patterns of age effects on disorders and disabilities that suggest psychiatric problems drop substantially by age 12, increase between ages 12 to 15, and drop again at age 16[4,22]. Nonetheless, it remains an important task to derive accurate estimates of prevalence of mental health problems in this age group in the national adolescent population.

Several large-scale national surveys of U.S. adults have included structured diagnostic interviews, for example, the Epidemiological Catchment Area Study (ECA)[25] the National Comorbidity Survey and the National Comorbidity Survey-Replication (NCS, NCS-R, age 15–55)[14,15,18]. Although the parallel National Comorbidity Survey – Adolescents (NCS-A,[18]) is a much-needed addition to the field, it has yet to yield estimates of psychiatric disorder among adolescents in the US. In the NCS, Kessler[14] reported that 12-month prevalence of mental disorders was consistently the highest in their youngest cohort, the 15–24 age-group. Gender differences were also reported in the NCS by Kessler[18,26].

One study that has recently incorporated screening items for mental health problems among adolescents in the United States is the NIMH Methods for the Epidemiology of Childhood and Adolescent Mental Disorders Study (MECA)[27]. This study used probability household samples of children aged 9 to 17 and their adult caretakers from four sites. The final sample included 1285 pairs of child respondent and adult caretaker. The screening instrument used in this study was a recently-developed instrument for diagnostic screening of children and adolescents, the Diagnostic Interview Schedule for Children (DISC-2.3)[28]. The DISC-2.3 is a highly structured diagnostic instrument, which screens for six categories of the most common mental disorders among children and adolescents (DSM-III-R)[29]: anxiety, affective, disruptive behavior, mood, substance use, miscellaneous (e.g., eating disorders), and psychotic disorders. This instrument has demonstrated good criterion validity with independent clinical diagnoses[30] and is a reliable tool for the screening of childhood mental disorders[28]. Almost one third of the youth sample (ages 9–17) met DSM diagnostic criteria for any disorder[28]. However, the MECA samples were small and local, and were not representative of the US population as a whole.

Another study that is in the process of developing estimates of child and adolescent (ages 4 – 17) mental health problems in the U.S., is the National Health Interview Survey (NHIS), an annual household survey with a nationally representative sample. In the 2001 and 2003 Supplements to the NHIS, the parent report version of the Strengths and Difficulties Questionnaire (SDQ) was included, and in the 2002 NHIS, a subset of items from this measure was included. Parent reports for approximately 9000 to 10000 children aged 4 – 17 were collected on five emotional and behavioral disorders[31]. The SDQ used in the NHIS includes five scales of five items each, assessing emotional symptoms, conduct problems, hyperactivity-inattention, peer relationship problems, and prosocial behavior, as well as items that tap impairment and burden[32]. The authors report estimates that approximately 5% of the non-institutionalized U.S. population aged 4 – 17 experienced severe emotional difficulties, with significant gender and age differences in the estimates of problems. Notably, however, these estimates are derived from *parent *reports, which for older adolescents especially, may underestimate the extent of these problems. It remains an important task, therefore, to derive self-reported estimates of mental health problems across the full range of adolescence.

Lucas and colleagues[33] refined the use of DISC diagnostic scales for mental disorders previously employed in the MECA study[27] by devising brief screening scales that can be used to identify those who are likely to meet diagnostic criteria in full assessment. Based upon secondary analysis of the MECA data, Lucas and colleagues applied logistic regression models with stem items from the DISC (i.e., questions asked of all respondents) as the independent variables and DSM-III-R diagnosis as the dependent variable. For each specific diagnosis, the DISC predictive scale was comprised of all the items that emerged as significant predictors of the diagnosis in the regression[33]. Two cut-off scores were derived, one pre-defined by a positive response to any gate item (i.e., those that suggest the need for further probing in a given area), the other constructed to maximize the positive prediction of the full diagnosis based on the sum of sensitivity and specificity. The final instrument contains 76 items, much reduced from the 206 items in the full DISC scales[33].

For the first time in the 2000 NHSDA[34], the DISC Predictive Scale (DPS) items were included and asked of more than 25000 adolescent respondents. This paper reports the prevalence of symptom clusters of psychiatric problems among adolescents in the U.S. population derived from positive screens using the DPS. Although such positive screens are not equivalent to diagnostic case ascertainment – because the sensitivity and specificity data suggest that positive screens for symptoms may be overestimated for clinically significant disorders – these data are important indicators of mental health problems among age, gender and racial/ethnic subgroups that have not been well-documented elsewhere, and such estimates can extend our understanding of the extent of need and inform prevention efforts[19].

## Method

### The Data

Prevalence and co-occurrence estimates of symptom clusters of specific psychiatric problems were derived from the 2000 National Household Survey on Drug Abuse (NHSDA)[34]. Specifically, we used the adolescent (12–17 years) sample from public use file (N = 19,430), which is a random sample of the total adolescent sample (N = 25,717)*. In the 2000 survey, a complete module of 22 items that approximates DSM-IV[36] criteria for dependence and abuse on major substances such as alcohol, nicotine, marijuana, cocaine and other drugs was included, making it possible to estimate the prevalence of clinically defined dependence for each substance. Also included was a comprehensive set of 71 mental health questions adapted from the DPS, which provided a unique opportunity to estimate the prevalence and co-occurrence of symptom clusters of major psychiatric problems among this critical age group in a large population sample.

The NHSDA sample is drawn using a multi-stage stratification procedure from the civilian, non-institutionalized U.S. population aged 12 or older. Computer-assisted face-to-face interviews were performed in the selected households. Of the 19,340 adolescent respondents in the public data, approximately half were female (49.3%). The racial/ethnic composition was 67% White, 14% Black, 14% Hispanic, and 6% other race or ethnicity. Table 1 presents the demographic characteristics of the 2000 NHSDA adolescent sample and comparison data for the US population aged 12–17 from the 2000 census[37]. Our sample deviates somewhat from the comparison population only in the distribution of adolescents in small- and non-metropolitan areas (probably due to differing Census and NHSDA sample frames).

**Table 1:** Characteristics of Adolescents Aged 12 – 17 in NHSDA and U.S. Population (Census 2000)

	U.S. Population	NHSDA Unweighted	NHSDA Weighted
**Age**	12 – 17	12 – 17	12 – 17
N	24,179,360	19,430	23,367,782
**Sex**			
Male (%)	51.4	50.7	51.2
Female (%)	48.6	49.3	48.8
**Race/Ethnicity**			
White (%)	63.4	66.7	65.5
Black (%)	14.4	13.5	14.3
Hispanic (%)	15.2	13.9	14.2
Other (%)	7.0	5.9	6.0
**Region**			
Northeast (%)	18.0	19.3	17.9
Midwest (%)	23.5	25.6	23.4
South (%)	35.4	32.2	35.6
West (%)	23.1	22.9	23.0
**Population Density**			
MSA (pop≥1 m)	43.5	39.0	43.6
MSA (pop < 1 m)	35.7	34.6	33.4
Not in MSA	20.8	26.4	23.1

### The Key Variables

DISC predictive scales (DPS) were used to calculate 14 psychiatric symptom clusters. By symptom cluster we refer to a group of symptoms derived from DSM-III-R (DISC 3.2) disorders without the criteria on impairment or duration of the symptom. To derive the score for each symptom cluster, we summed across the DPS items for each scale that were answered positively by the respondent (see Appendix for a listing of the items used for each scale Additional file: 1). The cutoff point for each symptom cluster is based on previous methodological studies of MECA[33] (see Table 2 for details). Three sub-scales in the NHSDA – elimination, panic and mania – were not included in the previous methodological study. Due to a lack of data on sensitivity and specificity thresholds for these scales, we used the maximum score (positive on all items) to define the symptom cluster for each to minimize over-estimation. Thus, a positive case of symptom cluster was derived only when a respondent met the predetermined cut-off for symptom items in the DISC predictive scales. The DPS symptom clusters measured in this way include seven anxiety problems, two affective problems; three disruptive-behavior problems, and two miscellaneous problems (*eating & elimination problems*). Four indexes of substance use disorders were derived using the modules for approximate diagnosis included in the NHSDA.

**Table 2:** Comparison of Item Numbers and Cutoffs for Each Subscale of DISC Predictive Scales in MECA and NHSDA

	Total	SiPh	SoPh	Agor	OAD	OCD	SAD	Eat	MDD	Adhd	ODD	CD	Elim	Man	Panic
**Study based on MECA^a^**													Not in previous report ^b^		
# items in full DISC	206	14	8	4	17	13	18	7	27	44	12	24			
# items in DPS	77^c^	7	5	3	7	7	8	3	9	13	7	9			
Optimal DPS cutoff		≥ 2	≥ 1	≥ 1	≥ 4	≥ 1	≥ 3	≥ 2	≥ 4	≥ 4	≥ 4	≥ 2			
Sensitivity		0.77	0.89	0.37	0.85	0.91	0.89	0.90	0.98	1.00	0.96	1.00			
Specificity		0.79	0.74	0.96	0.92	0.72	0.85	0.88	0.90	0.85	0.94	0.98			
**NHSDA 2000**															
# items in NHSDA	71	7	2	4	4	5	7	4	7	6	7	8	3	5	2
Proposed cutoff		≥ 3	≥ 2	≥ 2	≥ 3	≥ 3	≥ 4	≥ 2	≥ 5	≥ 4	≥ 4	≥ 2	= 3	= 5	= 2

Note: DISC = Diagnostic Interview Schedule for Children (v2.3); DPS = DISC Predictive Scales; MECA = Methods for the Epidemiology of Child and Adolescent Mental Disorders; SiPh= simple phobia, a.k.a. specific anxiety in DSM-IV; SoPh = social phobia; Agor = agoraphobia; OAD = overall anxious disorder, or general anxiety disorder in DSM-IV; OCD = obsessive-compulsive disorders; SAD = separation anxiety disorder; Eat = eating disorders; MDD = major depressive disorders; ADHD = attention-deficit/hyperactivity disorder; ODD = oppositional defiant disorder; CD = conduct disorders; Elim = elimination disorders; Man = Mania; Panic = Panic disorders.

a. From Lucas et al. 2001. Adjustment was made based on further data analysis and pilot studies of DPS by Lucas.

b. Three sub-scales in the 2000 NHSDA were not reported in previous methodological study. Due to lack of data on sensitivity and specificity for these scales, we used the maximum score (positive on all items) as the cutoff for the predictive diagnosis.

c. Excluding one question on substance use disorder, which was replaced by 19 items in NHSDA.

We further aggregated the DPS symptom clusters into indexes reflecting the presence of any cluster within each category (a*ny anxiety, any affective, any behavior*, and *any SUD*). Lastly, we created two variables, *any DPS cluster *and *≥4 DPS clusters*, to tap the prevalence of having any DPS cluster and the severe co-occurrence of multiple DPS clusters, respectively.

As described earlier, items included in the DPS were derived from the full set of DISC items by logistic regression models to predict DSM diagnoses. The number of gate items for each diagnosis-specific scale ranged from 3 to 7. Further, the cut-off points were established by "any gate item answered positively" and by the criterion that the "sum of sensitivity and specificity was maximized for the positive prediction of each specific diagnosis"[33]. Sensitivity ranged from 0.37 to 1.00, with most above 0.89; specificity ranged from 0.72 to 0.98, with most above 0.90. The subset of gate items on the final DPS scales has been demonstrated to identify with 100% accuracy those respondents who *don't *have a diagnosis, and further contingent items can be omitted without threat of missing a positive case[33]. Therefore, the DPS provides us with a useful and relatively reliable tool to identify those in the U. S. adolescent population at elevated risk for mental disorders.

However, it is important to keep in mind that sensitivity and specificity information may have different implications for different base rates since they are more or less inherent measurement properties of an instrument, but the predictive value also depends to a great extent on the actual probability or base rate of the measured symptom. In other words, the information may be more relevant or sensitive to the symptom with high prevalence in the population, but less relevant or informative for the symptom with very low prevalence in the population.

### Analytic Strategy

First, we estimated the prevalence for each of the 19 DPS clusters (including the 5 SUD clusters) for the total sample and for each subgroup, and then compared them within demographic categories. To avoid type-I errors in the estimates, we only report differences significant at the *p *< 0.01 level. As the NHSDA used a multiple-level sampling procedure, the standard errors for all prevalence rates were estimated by SUDAAN[38], a software package that uses Taylor Series linearization techniques to adjust for sample design effects. Next, we examined the rates of co-occurrence of clusters among demographic groups in two ways: the proportion of the total sample reporting two or more past-year DPS clusters, and the proportion of those with at least one DPS cluster. Last, we explored and identified significant demographic correlates of each cluster through application of multivariate logistic regression models.

## Results

### Prevalence Estimates

Twelve-month prevalence of symptom clusters of psychiatric problem was estimated using the DPS. As shown in Table 3, three out of five (58.1%) adolescents aged 12–17 years screened positive for at least one DPS cluster over the 12 months preceding the survey.

**Table 3:** Twelve-Month Prevalence of Psychiatric Symptom Clusters based on DISC Predictive Scale ^a ^Among U.S. Adolescents by Gender, Race/Ethnicity and Age (NHSDA 2000)

	**Total 12–17 **year old		By Sex				By Race/Ethnicity						By Age			
			
			Male		Female		White		Black		Hispanic		12–14		15–17	
	
	%	SE ^b^	%	SE	%	SE	%	SE	%	SE	%	SE	%	SE	%	SE
**Anxiety Clusters**																
Social phobia	**16.5**	0.32	**14.7**	0.45	18.4*	0.45	**15.1**	0.38	**22.2**	0.95	17.0*	0.94	**18.1**	0.48	14.8*	0.39
Separation anxiety	**7.3**	0.22	**5.5**	0.28	9.1*	0.33	**6.1**	0.25	**11.2**	0.79	9.1*	0.59	**9.9**	0.34	4.6*	0.26
Agoraphobia	**9.3**	0.24	**5.9**	0.28	12.9*	0.39	**7.5**	0.25	**13.7**	0.75	13.0*	0.84	**11.4**	0.39	7.2*	0.31
Panic disorder	**6.0**	0.19	**3.8**	0.23	8.3*	0.30	**5.9**	0.22	**7.2**	0.58	**5.9**	0.48	**6.2**	0.28	**5.8**	0.27
General anxiety	**15.0**	0.29	**11.4**	0.38	18.9*	0.43	**15.6**	0.36	**15.6**	0.83	13.2*	0.69	**14.4**	0.39	**15.7**	0.44
Specific phobia	**13.5**	0.28	**8.4**	0.32	18.9*	0.45	**11.7**	0.31	**20.0**	0.88	14.1*	0.78	**15.5**	0.40	11.4*	0.38
OCD	**14.4**	0.30	**11.8**	0.40	17.1*	0.47	**11.4**	0.31	**22.8**	0.97	17.7*	0.89	**15.2**	0.42	13.5*	0.43
*Any anxiety cluster*	**40.1**	0.41	**33.1**	0.56	47.4*	0.60	**36.8**	0.48	**50.8**	1.13	42.9*	1.17	**42.6**	0.58	37.5*	0.60
**Affective Clusters**																
Major depression	**12.1**	0.26	**7.9**	0.31	16.5*	0.42	**12.2**	0.31	**10.6**	0.69	**12.0**	0.68	**10.7**	0.37	13.5*	0.40
Mania	**3.1**	0.15	**2.7**	0.20	3.6*	0.21	**3.2**	0.19	**2.8**	0.35	**2.7**	0.31	**3.0**	0.21	**3.3**	0.21
*Any affect cluster*	**13.4**	0.28	**9.2**	0.34	17.9*	0.44	**13.6**	0.33	**11.7**	0.73	13.1*	0.70	**12.0**	0.39	14.9*	0.41
**Substance Use Disorders**																
Alcohol abuse ^c^	**3.3**	0.15	**3.4**	0.21	**3.3**	0.20	**3.8**	0.21	**1.9**	0.31	3.0*	0.35	**1.2**	0.13	5.4*	0.28
Alcohol dependent	**1.9**	0.12	**1.8**	0.17	**1.9**	0.18	**2.1**	0.16	**0.7**	0.18	1.9*	0.30	**0.6**	0.08	3.1*	0.24
Nicotine dependent	**4.1**	0.16	**3.7**	0.22	4.6*	0.24	**5.1**	0.22	**1.5**	0.24	3.2*	0.39	**1.7**	0.15	6.7*	0.30
Drug abuse ^c^	**2.0**	0.13	**2.1**	0.18	**2.0**	0.17	**2.1**	0.15	**1.7**	0.31	**2.6**	0.43	**1.0**	0.12	3.1*	0.22
Drug dependent	**2.4**	0.13	**2.6**	0.20	**2.2**	0.16	**2.5**	0.17	**1.7**	0.28	**2.3**	0.38	**0.9**	0.11	3.8*	0.24
*Any SUD*	**9.7**	0.25	**9.5**	0.34	**9.9**	0.35	**10.8**	0.33	**5.4**	0.50	9.1*	0.68	**4.0**	0.22	15.5*	0.45
**Disruptive-behavior Clusters**																
ADHD	**14.7**	0.29	**13.8**	0.41	15.6*	0.44	**13.8**	0.34	**18.3**	0.91	15.1*	0.82	**16.0**	0.44	13.3*	0.37
ODD	**27.1**	0.38	**27.9**	0.56	**26.2**	0.53	**27.8**	0.47	**26.4**	1.10	**24.9**	0.94	**27.2**	0.53	**27.0**	0.52
Conduct disorder	**11.5**	0.28	**12.8**	0.41	10.1*	0.39	**10.9**	0.32	**12.4**	0.79	**12.2**	0.73	**9.3**	0.34	13.6*	0.41
*Any beh. cluster*	**36.3**	0.42	**37.1**	0.59	**35.5**	0.60	**36.3**	0.51	**38.3**	1.15	**34.7**	1.09	**36.1**	0.59	**36.6**	0.55
**Other Disorder Clusters**																
Eating disorder	**6.1**	0.19	**3.7**	0.23	8.7*	0.32	**5.9**	0.24	**6.5**	0.57	**6.4**	0.50	**5.5**	0.28	6.8*	0.30
Elimination	**0.2**	0.05	**0.3**	0.08	**0.2**	0.05	**0.3**	0.06	**0.2**	0.09	**0.2**	0.06	**0.2**	0.06	**0.3**	0.07
***Any DPS Cluster***	**58.1**	0.42	**54.8**	0.56	61.6*	0.59	**56.7**	0.51	**62.8**	1.15	58.8*	1.17	**56.6**	0.59	59.6*	0.58

* *p *< 0.01 in Chi-square test of gender, race or age differences. ^a ^DISC = Diagnostic Interview Schedule for Children, See appendix Table A; ^b ^All standard errors are estimated by SUDAAN, which takes multi-level sampling effects into consideration. ^c. ^Abuse only without dependence.

#### Gender

Although female adolescents usually report lower rates of substance use, the prevalence of SUDs for females in this national sample is as high as that of males. Females were more likely to be nicotine dependent (although males reported more nicotine use than females). Compared to males, females reported higher rates of anxiety, affective, and eating problems, and lower levels of the elimination problems.

#### Age

Estimates of having any DPS psychiatric symptom cluster were slightly higher for late vs. early adolescents, 59.6% vs. 56.6%, and resulted mainly from age differences in SUD. Late adolescents (ages 15–17) reported higher rates of SUD and affective clusters; younger adolescents (age 12–14) report higher rates of anxiety clusters than older adolescents (age 15–17); and no age differences emerged in rates of behavioral clusters with the exception of ADHD, which was more prevalent among the younger group.

#### Race/Ethnicity

Blacks reported more DPS psychiatric symptom clusters than Whites and Hispanics. As would be expected from the literature, Blacks reported lower use of licit and illicit substances[39], and exhibited lower estimates of SUD. Blacks also appear to have relatively high risk for anxiety clusters, compared to other ethnic groups. Rates of OCD clusters among Blacks were twice as high as among Whites, and rates for specific anxiety and agoraphobia clusters also approached this degree of difference.

### Co-Occurrence

Fifty-eight percent of the adolescent sample met risk-identification criteria for at least one psychiatric symptom cluster in the 12 months preceding the survey. Table 4 shows that 37.7% of the sample met criteria for two or more co-occurring symptom clusters, and 17% reported 4 or more clusters. Of those with at least one identified cluster, almost two-thirds screened positive for an additional one or more clusters: 20.9% reported two, 14.6% reported three, and 29.4% reported four or more (i.e., severe comorbidity). Thus, co-occurrence of mental health problems in this group appears quite high and deserving of more attention. In general, females had higher rates of severe comorbidity than males, with about one third of females and one quarter of males having at least one cluster screening positive for four or more. Black adolescents reported higher rates of comorbid clusters than any other ethnic group; and younger adolescents had a slightly higher rate of comorbidity than their older counterparts.

**Table 4:** Co-occurrence of 12-Month Psychiatric Symptom Clusters (including SUD) among Adolescents with any DPS Symptom Cluster, by Gender, Age and Race/Ethnicity

*No. of 12-mth clusters*	0	1	2	3	4+
**Among total sample (N = 19,430)**					
Total (%)	41.9	20.4	12.2	8.5	17.1
Male	45.2	21.5	12.4	7.9	13.0
Female	38.4	19.2	11.9	9.1	21.4
White	43.3	20.8	11.9	8.1	15.9
Black	37.2	18.7	13.5	9.1	21.4
Hispanic	41.2	20.0	12.1	8.5	18.2
Others	39.9	20.3	11.6	10.4	17.7
Age 12–14	43.4	19.3	12.0	8.2	17.1
Age 15–17	40.4	21.5	12.3	8.8	17.1
**Among those with any DPS cluster **(N = 11,228)					
Total (%)	--	35.1	20.9	14.6	29.4
Male	--	39.2	22.6	14.4	23.8
Female	--	31.2	19.4	14.7	34.7
White	--	36.7	21.0	14.4	27.9
Black	--	29.8	21.6	14.5	34.1
Hispanic	--	34.0	20.5	14.5	31.0
Others	--	33.8	19.3	17.3	29.5
Age 12–14	--	34.1	21.2	14.5	30.2
Age 15–17	--	36.1	20.6	14.7	28.6

### Demographic Correlates of Psychiatric Symptom Clusters

To further understand the relationship of gender, ethnicity, age and other demographic factors on the estimated prevalence of 12-month psychiatric symptom clusters and co-occurrence, we conducted a series of multiple logistic regression analyses, estimated by SUDAAN, to explore the significant demographic correlates for each domain of clusters, for any DPS cluster, and for severe comorbidity (4 or more clusters). Table 5 presents the adjusted odds ratios for each demographic factor in each of these LOGIT models.

**Table 5:** Odds Ratios of Demographic Correlates of 12-month Psychiatric Symptom Clusters Based on Logit Models

Correlates	*Any anxiety cluster*		*Any affective cluster*		*Any SUD*		*Any behavior cluster*		*Any DPS symptom cluster*		*4+ clusters*	
	
	OR	95% CI	OR	95% CI	OR	95% CI	OR	95% CI	OR	95% CI	OR	95% CI
Sex												
Male	0.55*	0.51–0.59	0.47*	0.42–0.52	0.98	0.88–1.10	1.08*	1.00–1.16	0.76*	0.71–0.81	0.56*	0.51–0.61
Female	1.00	--	1.00	--	1.00	--	1.00	--	1.00	--	1.00	--
Age												
12–13	1.00	--	1.00	--	1.00	--	1.00	--	1.00	--	1.00	--
14–15	0.85*	0.79–0.93	1.20*	1.07–1.35	3.96*	3.23–4.85	1.17*	1.08–1.26	1.16*	1.05–1.23	0.96	0.87–1.06
16–17	0.75*	0.68–0.81	1.34*	1.19–1.51	8.01*	6.54–9.83	1.03	0.95–1.11	1.12*	1.04–1.22	0.95	0.85–1.06
Race/ethnicity												
White	1.00	--	1.00	--	1.00	--	1.00	--	1.00	--	1.00	--
Black	1.67*	1.50–1.85	0.86	0.73–1.01	0.43*	0.35–0.54	1.08	0.97–1.21	1.24*	1.10–1.38	1.32*	1.13–1.53
Hispanic	1.20*	1.07–1.35	0.97	0.84–1.12	0.79*	0.64–0.98	0.97	0.87–1.09	1.06	0.95–1.18	1.11	0.97–1.28
Others	1.26*	1.06–1.49	1.24	0.98–1.56	0.79	0.57–1.09	1.09	0.91–1.30	1.14	0.96–1.35	1.13	0.89–1.44
Family income ($)												
0–19,999	1.32*	1.17–1.49	0.99	0.84–1.17	1.49*	1.22–1.82	1.04	0.93–1.17	1.19*	1.06–1.34	1.52*	1.31–1.78
20,000–39,999	1.33*	1.20–1.48	0.97	0.85–1.12	1.52*	1.28–1.81	1.11*	1.00–1.24	1.24*	1.12–1.38	1.48*	1.28–1.71
40,000–74,999	1.04	0.94–1.15	0.96	0.83–1.10	1.21*	1.03–1.42	1.04	0.94–1.14	1.07	0.97–1.18	1.15*	1.00–1.31
75,000 +	1.00	--	1.00	--	1.00	--	1.00	--	1.00	--	1.00	--
Population density												
MSA 1 million+	0.90*	0.82–0.99	0.87*	0.76–0.99	0.84*	0.72–0.99	1.05	0.95–1.15	0.96	0.87–1.06	0.88*	0.78–1.00
MSA <1 million	0.98	0.90–1.08	0.95	0.84–1.07	0.92	0.79–1.06	1.05	0.96–1.15	1.02	0.93–1.11	0.99	0.87–1.12
Non-MSA	1.00	--	1.00	--	1.00	--	1.00	--	1.00	--	1.00	--
U.S. Born	0.91	0.78–1.06	0.91	0.75–1.11	1.65*	1.21–2.25	1.41*	1.19–1.67	1.09	0.94–1.26	1.17	0.96–1.43
School dropout	0.86	0.67–1.10	0.95	0.67–1.34	2.20*	1.63–2.97	1.01	0.79–1.30	1.03	0.81–1.31	1.22	0.88–1.69
**Models with sex-by-age interaction ¶**												
Sex *Age interaction, (vs. female, age 12–13)												
Male, 14–15	0.73*	0.62–0.86	0.61*	0.48–0.79	0.75	0.49–1.15	0.86	0.73–1.02	0.76*	0.65–0.89	0.76*	0.61–0.95
Male, 16–17	0.67*	0.56–0.80	0.62*	0.49–0.80	0.89	0.60–1.34	0.89	0.75–1.05	0.77*	0.65–0.91	0.75*	0.59–0.94

* *p *< .05 (two tailed test) – Models estimated by SUDAAN with correction of design effects;

¶ Shows the interactive effects only with presenting other correlates.

#### Gender

In support of the prevalence estimates reported above, males had a lower likelihood of reporting any DPS cluster. Specifically, they were less likely to report any anxiety or affective cluster, or severe co-occurrence. However, males were more likely to have a significantly elevated chance of attention deficient and behavior problems.

#### Age

We analyzed the age effect on symptom clusters in three groups, early adolescent (ages 12–13), middle-adolescent (ages 14–15), and late adolescent (ages 16–17). Whereas the middle adolescents were more likely to report either any DPS symptom cluster or disruptive-behavior cluster, there were considerable variations in the odds ratios of reporting clusters across age groups. For example, the younger adolescents were more likely to meet criteria for an anxiety cluster than the older adolescents, whereas the older adolescents were more likely to do so for an affective cluster. Middle-adolescents had the highest risk for attention deficient and behavioral problems, suggesting the transitory and developmental nature of these problems. Confirming the wealth of data in the literature, the odds of SUD increased with age: middle-adolescents were almost four times as likely, and late adolescents eight times as likely as early adolescents to report any SUD.

#### Gender-Age interactions

A number of significant gender-age interactions appeared in the models predicting any DPS symptom cluster, anxiety clusters, affective clusters, and severe co-occurrence (see models in the lower portion of Table 5). Generally, compared to the youngest females (12–13), middle and late adolescent males (14–17) had lower odds for each of these problems. Taken together with the findings of prevalence reported earlier, it seems that the youngest females are at the highest risk for these clusters and for the more severe levels of comorbidity problems.

#### Race/Ethnicity

Black adolescents are at greater risk than White not only for any DPS symptom clusters but also for severe co-occurrence, and they are at higher risk for anxiety problems as well. Indeed, all minority groups in this sample were more likely to report anxiety problems than Whites. However, Blacks alone are at increased risk for co-occurrence once they have met criteria for any one symptom cluster.

#### Family Income

Adolescents from families with lower incomes are more likely to meet criteria for at least one psychiatric symptom cluster and to be at risk for severe comorbidity compared to their more affluent peers. This relationship held for anxiety cluster and SUD, although for SUD there was increased risk for all adolescents save those from the wealthiest families (income ≥ $75 k).

#### Other Demographics

Interestingly, compared to those from non-metropolitan areas, adolescents from the largest metropolitan areas were at reduced risk for most psychiatric problems, including anxiety and affective domains, SUD and co-occurring problems. US-born adolescents had higher risk for SUD and disruptive behavior problems in comparison with immigrant adolescents. School dropouts were at higher risk for SUD than those currently enrolled in school.

## Discussion

Although the data from NHSDA are not sufficient to reach any clinical diagnosis, these unique data offer us important descriptive information on the epidemiology of psychiatric symptoms and their variations in gender and ethnic groups among the U.S. adolescent population. Even a conservative interpretation of these estimates from a nationally representative sample of the U.S. adolescents suggests that adolescent mental health problems and related co-occurrence may be more serious than previously believed. Almost three out of five US adolescents aged 12–17 screened positive for a symptom cluster of specific psychiatric problem using the DPS scales in the 12 months prior to the interview. More than one third reported a disruptive-behavior problem and a slightly larger proportion reported an anxiety problem. Approximately one out of every eight adolescents reported an affective problem, and one in ten had a substance use disorder. Although the high prevalence may be inflated by behavioral symptoms that are likely to be significantly reduced with maturation, these estimates should raise serious concerns about the mental health status of U.S. adolescents. Although these symptom clusters do not fully emulate the diagnostic criteria of clinical disorders, the previous study of specificity and sensitivity of DPS suggested that these symptom clusters have good predictive validity for a disorder ascertained using the DISC[33]. Parenthetically recent epidemiological reports of 12-month DSM-IV rates of psychiatric disorders from the World Mental Health 2000 initiative among adults in European countries have found they are far lower than the U.S. national rates measured with the same DSM-IV diagnostic protocol[40]. In the Oregon Adolescent Depression Project, the concept of sub-threshold psychiatric conditions was introduced to monitor the potential mental health problems among adolescents[41], which is very similar to the symptom cluster of psychiatric problem proposed in this study. They reported that, of the 1704 adolescents in the study, 52.5% had at least one sub-threshold disorder; of those 40% had also experienced a comorbid sub-threshold condition, and 30% had a second comorbid condition[41]. Based on this updated information, our estimates of high prevalence of psychiatric symptom clusters among U.S. adolescents based on the largest national household survey are not farfetched. The key is how we interpret these data and how we use the information. For example, it is possible to view these as estimates of adolescents "at-risk" rather than as ascertained cases since the items used for the estimates were derived from psychiatric symptoms used in the actual DISC diagnostic modules. Even in instances where the number or pattern of symptoms are sufficient for a "screened positive" but not sufficient to constitute a recognized psychological disorder, the evidence suggests that many of these children do indeed have mental health problems [42], and are in need of further, expert assessment. We acknowledge, however, that surveys such as the NHSDA rely upon the anonymity of their respondents to ensure accurate collection of sensitive data; for this reason, such referral is impossible.

Our results show that girls tend to have a higher rate of mental health problems than boys, which is consistent with the literature [15-17] and with estimates for the adult population as reported in the NCS study[14]. However, contradicting findings from community or treatment samples, prevalence of disruptive-behavior problems was similar among boys and girls; additionally, in the present study, there were no gender differences for SUD, in contrast to the findings from the NCS for the US population aged 15–24, where males had a significantly higher rate of SUD than females. Our data also contradict previous findings in this area: although numerous national surveys consistently report lower rates of substance *use *among females[17,39], we found that females were equally likely to be dependent upon or abuse substances, suggesting the possibility that once they begin use, females are more at risk for developing an SUD.

Another contradictory finding emerged concerning our estimates of disruptive behavior. In treatment samples, males reported higher rates of disruptive-behavior problems; in our study, however, estimates of behavior problems suggest similar rates for male and female adolescents in the general population.

As to the racial/ethnic differences, Blacks reported a higher rate of psychiatric problems than other groups, including anxiety problems. These findings are in marked contrast to those reported from the NCS, where no racial difference was found in the prevalence of anxiety disorders, including simple phobia and agoraphobia[14]. However, the data for adolescents presented here are similar to those reported from the ECA study[43].

It is interesting to notice the reduced risk of mental health problems in large metropolitan areas, since some assume that large metropolitan areas may have more stressors and mental health problems, and are the areas with greater need for mental health services. The finding here raises the prospect that mental health service delivery to the rural areas (e.g. mobile clinics, tele-support network, etc.) should become a priority in health service planning.

Evidence emerging from epidemiological studies suggests high levels of comorbid mental health problems among children and adolescents [44-46]. The proportion of adolescents in the present sample who meet criteria for more than one psychiatric symptom cluster is astonishingly high – about two-thirds of those with one symptom cluster were at risk for other symptom clusters, and half of these met criteria for four or more symptom clusters (see Table 4). Such high estimates of co-occurring mental health problems are surely deserving of attention and raise important issues of providing appropriate treatment and preventive interventions. We identified a number of risk factors for severe psychiatric comorbidity including Black race, lower family income, and being a younger female (12–13 years old). Previous studies of the relationship between psychiatric problems and substance use in adolescents suggests an approximately linear relationship between the intensity of use and the likelihood of having a mental problem, especially conduct disorder [47-49]. Thus this issue remains an important topic for future studies, especially with nationally-representative samples.

### Limitations

There are a number of limitations that might affect the findings reported in this paper. First, the NHSDA is a cross-sectional study that relies solely upon retrospective self-reports of symptoms and behaviors; therefore, the reliability of past 12-month recall of adolescent mental health symptoms needs further verification.

Second, we reiterate that the DISC Predictive Scales used to derive prevalence of psychiatric symptom clusters identify positive screens for disorders – they are not clinical diagnoses, and should be considered as indicators of *elevated risk *for psychiatric disorders. Compared to DSM-IV criteria, these scales do not have the required duration or replication measures (as DPS was developed with DISC criteria). Even with this limitation, however, these data speak to important patterns of age, gender and ethnic differences in the prevalence of mental health problems deserving of further study.

Third, the DPS does not have the impairment or severity measures of each symptom that are frequently used in clinical diagnoses. Estimates based on symptoms alone can vary drastically from those derived using impairment criteria[19]. Using these additional criteria increases the likelihood that the ascertainment in a field interview would more closely resemble a clinically ascertained case in terms of severity. However, collecting this level of information about each respondent was and is not feasible for the type of survey used in our study. It should be noted that using additional impairment criteria does not necessarily affect caseness estimates, and the criteria we have used for case (of at-risk) identification have shown good sensitivity and specificity when compared to the use of a real diagnostic interview (e.g., DISC). Moreover, children who meet criteria for psychiatric disability without meeting full DSM-IV criteria for diagnosis can be considered as having significant psychiatric problems[42].

Finally, despite the use of anonymous and computer-assisted interview (CAI) techniques, household surveys tended to yield lower rates of substance use compared to school-based surveys such as Monitoring the Future[39] and Youth Risk Behavior Surveillance Survey[50]. Youth matched for high-school grade and age in the NHSDA reported lower rates of substance use than those from other school-based surveys[51]. Therefore, although the estimated rates of symptoms of mental health problems in our sample of adolescents are very high, the possibility of under-reporting in NHSDA is still of an unknown degree and needs further studies to verify its extent.

### Clinical Implications

Although the United States has a well-established system for monitoring the pattern of drug use in the general adolescent population, through both school-based surveys[39] and the household survey (NHSDA), there is no system existing for estimating rates of substance use disorders and mental disorders in the population on an ongoing basis and among various socio-demographic subgroups. The current estimates of the prevalence and co-occurrence of adolescent psychiatric problems, especially the dramatic gender and ethnic variations in different psychiatric problem domains, may provide clinicians with both a heightened alert and a useful tool with which to identify potential mental health problems in the general adolescent population, which traditionally has been biased by extensive reliance on results from treatment studies.

Black and poor adolescents reported mental health problems more frequently than did those from other groups; unfortunately, both Black and poorer adolescents are less likely to receive diagnosis or treatment for mental health problems[21,52]. Clearly, the present findings strongly suggest a need not only for increased efforts to identify and treat adolescents with psychiatric problems but also to design and implement effective preventative and treatment strategies for the most needy subgroups.

Integrating the DISC predictive scales in the NHSDA (now NHSDUH) would provide an excellent mechanism for including an expanded set of questions designed to systematically monitor and assess substance use disorders and psychiatric problems. Perhaps by using alternating numbers of diagnostic modules per survey iteration, NHSDUH can regularly include the mental health module as it did for SUDs. Such regular inclusion of these modules will allow for an examination of changes over time, an important goal for estimating trends in the prevalence of mental health problems. We recommend that the Substance Abuse and Mental Health Service Administration study and institute the expansion of DPS in future waves so that researchers and policy makers can continuously monitor the trends and patterns in adolescent mental health problems that will provide essential information for the development of more effective prevention and intervention programs.

## Competing interests

The author(s) declare that they have no competing interests.

## Authors' contributions

• **Dr. Kevin Chen **initiated the paper plan, performed data analysis and initial literature review, and wrote up the draft of the paper.

• **Dr. Ley Killeya-Jones **participated in planning of the paper, performed most literature search and review, wrote-up the introduction and discussion, and polished the entire paper.

• **Dr. William Vega **participated in planning of the paper, defining the concept of symptom cluster of psychiatric problem instead of traditional "disorder", provided key literature and conceptual support, and editing and polishing the entire paper.

## Note

* Due to concerns with confidentiality, we were unable to have released to us the entire NHSDA sample by the Substance Abuse and Mental Health Service Administration (SAMHSA).[35]

## Supplementary Material

Additional File 1 "appendix_items.doc" Click here for file
