# Supplementary material for: Prevalence and co-occurrence of psychiatric symptom clusters in the U.S. adolescent population using DISC predictive scales
Source: Clin Pract Epidemiol Ment Health. 2005 Oct 28;1:22. doi: 10.1186/1745-0179-1-22 (PMC1298317; doi:10.1186/1745-0179-1-22)
Supplement: Additional File 1 — "appendix_items.doc" [file 1745-0179-1-22-S1.doc]

**APPENDIX**

Items comprising the subscales of Disc Predictive Scales used in the NHSDA 2000 ^35^.

All items (except where noted below) began with the stem, “***During the past 12 months***… “ and used the answer key, *yes, no,* or *don’t know/refused to answer*.

*Social Phobia (2 items)*

- have you *often* felt very nervous or uncomfortable when you have been with a group of children or young people — say, like in the lunchroom at school or at a party?
- have you *often* felt very nervous when you’ve had to do things in front of people?

*Separation Anxiety Disorder (7 items)*

- has there been a time when you *often* wanted to stay at home and not go to school or other places without your mother or father?
- have you *often* had headaches or stomachaches or felt like you would throw up when you couldn’t be with your mother or father?
- have you had a lot of bad dreams or nightmares?
- have you *often* wanted to have your mother or father near you before you could fall asleep?
- have you kept worrying that your mother or father might go away and never come back?
- if your mother or father has to go out some place without you do you *often* get very upset or beg him or her not to leave?
- if you were away from home without your mother or father for several days in a row --like staying with friends or relatives or going to camp, did you get very upset or very homesick because you missed your mother or father?

*Agoraphobia (4 items)*

- have you *often* been afraid to go out of the house by yourself?
- have you *often* felt afraid of being in crowded places?
- have you *often* been afraid of traveling in cars or on buses or trains?
- have you *often* felt afraid of being on bridges or in tunnels?

*Panic Disorder (2 items)*

- have you had an attack when all of a sudden you felt very afraid or strange?
- have you had a time when you suddenly felt that you were suffocating or you couldn’t breathe?

*General Anxiety Disorder (4 items)*

- have you *often* worried a lot *before* you were going to play a sport or game or do some other activity?
- have you had a lot of headaches?
- have you had a lot of other aches and pains?
- have you *often* been very tense, or found it hard to relax?

*Simple Phobias (7 items)*

- have you been very afraid of dogs, birds, snakes, insects, or any other animals?
- have you been very afraid of being high up or in a high place?
- have you been very afraid of being in the dark?
- has seeing a needle or getting a shot made you much more afraid than other people?
- has the sight of blood or cuts made you very afraid?
- have you been very afraid of being on a bridge or in a tunnel or on a highway?
- have you been very afraid of being in an elevator or on an escalator?

*Obsessive Compulsive Disorder (5 items)*

- have you *had* to count things over and over again, or make yourself do things a certain number of times?
- was there a time when you washed your hands or body over and over again or changed your clothes many times each day because you thought they were dirty?
- have you *often* felt you should check on things over and over again? For example, checking that the front door is locked, that the stove is turned off, or that something else was done even though you knew it had been done?
- have you *often* worried over and over again that things you touch are dirty or have germs?
- have you had any other thoughts that kept coming back into your mind over and over again that you couldn’t get rid of?

*Eating Disorders (4 items)*

- has anyone worried that you were much too thin?
- have you *often* felt bad about yourself because you thought you were fat or overweight?
- have there been times when you thought about food or about eating *almost all the time?*
- [An eating binge is when someone stuffs themselves with a whole lot of food in a short time — like several whole pizzas or a whole chocolate cake or several containers of ice cream — and they don’t seem to be able to control how much they eat]*. During the past 12 months,* have you had an eating binge like this?

*Elimination Disorder (3 items)*

- have you wet the bed at night?
- have you wet your pants during the day?
- have you soiled yourself? By “soiled,” we mean you had a bowel movement and pooped in your pants, or on the floor, or somewhere *not in the toilet*.

*Depression (7 items)*

- has there been a time when nothing was fun for you and you just weren’t interested in anything?
- has there been a time when you had less energy than you usually do?
- has there been a time when you felt you couldn’t do anything well or that you weren’t as good-looking or as smart as other people?
- has there been a time when you thought seriously about killing yourself?
- have you tried to kill yourself?
- has there been a time when doing even little things made you feel really tired?
- has there been a time when you couldn’t think as clearly or as fast as usual?

*Mania/Manic Depression (5 items)*

- [Everyone gets in a bad mood sometimes. But some people feel grouchy or angry or irritable most of the time for several days in a row. They get *really* annoyed when other people interrupt them or don’t agree with everything they say. During the past 12 months, that is since DATEFILL,] have you had a time when you were grouchy or angry like this?
- has there been a time when you felt *very restless***,** so that you had to keep walking around or be on the move all the time?
- has there been a time when you *talked too much or too quickly*?
- has there been a time when you thought you had special abilities or powers which made you stronger, smarter, or better than most other people?
- has there been a time when you *often* felt like your *mind was racing too quickly* from one thought to another?

*ADHD (6 items)*

- have you *often* had trouble keeping your mind on what you are doing for more than a short time?
- have you *often* forgotten what you are supposed to be doing or what you had planned to do?
- have you *often* found it hard to keep your mind on what you are doing when other things are going on?
- have you taken medication for being overactive, being hyperactive, or having trouble paying attention?
- have you *often tried not* to do things where you would need to pay attention for a long time?
- have you *often* made a lot of mistakes because it’s hard for you to do things carefully?

*Oppositional Defiant Disorder (7 items)*

- have you gotten even with people by doing things like hurting them, messing up their things, or telling lies about them?
- have you refused to do what your parents or teachers told you to do?
- have you been grouchy or easily annoyed?
- have you done mean things to people on purpose?
- have you blamed someone else for your mistakes or for things you did that you shouldn’t have done?
- have you done things just to annoy people or make them mad?
- have people complained because you swore or used dirty language?

*Conduct Disorder (8 items)*

- have you been expelled from school for misbehavior -- that is, have you been told you could never go back to that school at all?
- have you shoplifted -- that is, stolen something from a store when you thought no one was looking?
- have you lied to get money or something else you wanted?
- have you snatched someone’s purse or jewelry?
- have you broken something or messed up some place on purpose, such as breaking windows, writing on a building, or slashing tires?
- have you stolen from anyone else when they weren’t around or weren’t looking?
- have you been physically cruel to an animal and hurt it on purpose?
- have you broken into a house, a building, or a car?
